# Mitochondrial dynamics as a novel treatment strategy for triple‐negative breast cancer

**DOI:** 10.1002/cam4.6987

**Published:** 2024-02-09

**Authors:** Yuechen Wang, Narumi Harada‐Shoji, Narufumi Kitamura, Yuto Yamazaki, Akiko Ebata, Masakazu Amari, Mika Watanabe, Minoru Miyashita, Hiroshi Tada, Takaaki Abe, Takashi Suzuki, Kohsuke Gonda, Takanori Ishida

**Affiliations:** ^1^ Department of Breast and Endocrine Surgical Oncology Tohoku University Graduate School of Medicine Sendai Japan; ^2^ Department of Medical Physics, Graduate School of Medicine Tohoku University Sendai Japan; ^3^ Department of Pathology Tohoku University Graduate School of Medicine Sendai Japan; ^4^ Department of Breast Surgery Tohoku Kosai Hospital Sendai Japan; ^5^ Department of Pathology Tohoku Kosai Hospital Sendai Japan; ^6^ Division of Nephrology, Endocrinology and Vascular Medicine Tohoku University Graduate School of Medicine Sendai Japan; ^7^ Department of Medical Science Tohoku University Graduate School of Biomedical Engineering, Tohoku University Sendai Japan; ^8^ Department of Clinical Biology and Hormonal Regulation Tohoku University Graduate School of Medicine Sendai Japan; ^9^ International Center for Synchrotron Radiation Innovation Smart (SRIS) Tohoku University Sendai Japan

**Keywords:** breast cancer, drug resistance, mitochondria, mitophagy

## Abstract

**Introduction:**

Triple‐negative breast cancer (TNBC), recognized as the most heterogeneous type of breast cancer (BC), exhibits a worse prognosis than other subtypes. Mitochondria dynamics play a vital role as mediators in tumorigenesis by adjusting to the cell microenvironments. However, the relationship between mitochondrial dynamics and metabophenotype exhibits discrepancies and divergence across various research and BC models. Therefore, this study aims to explore the role of mitochondrial dynamics in TNBC drug resistance and tumorigenesis.

**Methods:**

The Wst‐8 test was conducted to assess doxorubicin sensitivity in HCC38, MDA‐MB‐231 (TNBC), and MCF‐7 (luminal). Confocal microscopy and FACS were used to quantify the mitochondrial membrane potential (ΔφM), mitophagy, and reactive oxygen species (ROS) production. Agilent Seahorse XF Analyzer was utilized to measure metabolic characteristics. Dynamin‐related protein‐1 (DRP1), Parkin, and p62 immunohistochemistry staining were performed using samples from 107 primary patients with BC before and after neoadjuvant chemotherapy (NAC).

**Results:**

MDA‐MB‐231, a TNBC cell line with reduced sensitivity to doxorubicin, reduced ΔφM, and enhanced mitophagy to maintain ROS production through oxidative phosphorylation (OXPHOS)‐based metabolism. HCC38, a doxorubicin‐sensitive cell line, exhibited no alterations in ΔφM or mitophagy. However, it demonstrated an increase in ROS production and glycolysis. Clinicopathological studies revealed that pretreatment (before NAC) expression of DRP1 was significant in TNBC, as was pretreatment expression of Parkin in the hormone receptor‐negative group. Furthermore, low p62 levels seem to be a risk factor for recurrence‐free survival.

**Conclusion:**

Our findings indicated that the interplay between mitophagy, linked to a worse clinical prognosis, and OXPHOS metabolism promoted chemotherapy resistance in TNBC. Mitochondrial fission is prevalent in TNBC. These findings suggest that targeting the unique mitochondrial metabolism and dynamics in TNBC may offer a novel therapeutic strategy for patients with TNBC.

## 1 INTRODUCTION

Breast cancer (BC) is the most frequently diagnosed malignant tumor among women globally and ranks as the second leading cause of mortality.^1^ Typically, it is classified based on hormone receptor (HR) expression, specifically including estrogen receptor (ER) and progesterone receptor (PR), as well as the presence of human epidermal growth factor receptor 2 (HER2). However, approximately 10%–20% of BC lacks expression of ER, PR, and HER‐2, categorizing it as triple‐negative breast cancer (TNBC), which is one of the most heterogeneous subtypes associated with an unfavorable prognosis.^2^ Some novel targets, such as immune checkpoint inhibitors, have shown improved response rates when used in combination with anthracycline and taxane‐based chemotherapy. Nevertheless, early recurrence continues to significantly worsen patient prognosis.^3^, ^4^ Therefore, TNBC urgently requires innovative and more potent treatment strategies.

The discovery of the Warburg effect, where cancer cells maintain aerobic glycolysis even in the presence of sufficient oxygen, has led to the widespread adoption of 2‐deoxy‐2‐[fluorine‐18]fluoro‐D‐glucose positron emission tomography (FDG‐PET) combined with computed tomography for cancer detection.^5^, ^6^ Owing to its quicker assessment of tumor size than conventional imaging and its sensitivity in indicating chemotherapy efficacy, such as evaluating neoadjuvant chemotherapy (NAC) response during BC treatment, FDG‐PET is the preferred imaging method for assessing chemotherapy efficacy.^7^ However, the persistence of cancer cells, even without FDG uptake following NAC, implies that not all cancers exclusively rely on aerobic glycolysis.^8^, ^9^, ^10^, ^11^ Studies have revealed the survival of patients with BC with minimal FDG uptake, indicating that BC cells utilize other metabolic pathways.^12^

Glutamine metabolism, the pentose phosphate pathway, hexosamines, amino acids, and lipids have been observed to be closely related to oxidative phosphorylation (OXPHOS), providing support for cell proliferation.^9^, ^13^ In our previous research, we revealed the impact of metabolic products of invasive ductal carcinoma on pathways involving thymidine, alanine, asparagine, glutamine, arginine, and proline.^9^, ^14^

Mitochondrial dynamics, encompassing fusion, fission, and mitochondrial autophagy processes, constitute fundamental components of the tumor signaling pathway and are closely associated with mitochondrial metabolism, ensuring cellular adaptability.^15^ Studies have explored variations in the expression of mitochondrial dynamics within TNBC.^16^, ^17^ However, the relationship between mitochondrial dynamics and metabophenotype exhibits discrepancies and divergence across various research and BC models.^17^, ^18^ Mitophagy, a process involving the selective autophagy of mitochondria, maintains intracellular environment stability.^19^

The most well‐established pathway is initiated by the phosphatase and tensin homolog (PTEN) and tension protein homolog (PINK1), leading to the activation of the putative kinase 1 (PINK1)/Parkin pathway. This pathway is identified post‐mitochondrial division mediated by DRP1 and has been observed in several cancers.^5^, ^20^, ^21^, ^22^, ^23^ As the main drug in the classic formula for NAC treatment, doxorubicin is widely used and has remarkable effects, but it also has widespread drug resistance problems.^24^, ^25^ In addition, we also got some inspirations on mitochondrial metabolism from the limitations of FDG‐PET. We hypothesize that drug‐resistant TNBC may mainly rely on mitochondrial metabolism and active mitochondrial dynamics (such as mitochondrial fission and mitophagy) to evade drug effects and FDG‐PET tracking. Therefore, this study designs first using luminal, TNBC cell lines, and corresponding cell models under doxorubicin treatment for the aim of exploring mitochondrial metabolism and mitophagy and subsequently exploring the relationship between them and doxorubicin resistance in NAC through clinical pathology studies.

## 2 METHODS

### 2.1 Cell lines and culture

To demonstrate the specificity of TNBC among all subtypes, we selected the intracavitary cell line MCF‐7, which exhibits a better prognosis and reduced sensitivity to chemotherapy, as the subtype control. TNBC cell lines, including MDA‐MB‐231, HCC38, and MCF‐7, were obtained from the American Type Culture Collection (ATCC; Manassas, VA, USA). RPMI‐1640 medium (Gibco BRL, Grand Island, NY, USA) supplemented with 10% fetal bovine serum (FBS; Cosmo bio, USA) and 100 μg/mL penicillin/streptomycin (Gibco BRL, Grand Island, NY, USA) was used for cell culture. Cells were cultured under conditions of 37°C and 5% CO_2_.

### 2.2 Cell proliferation assay

To identify cells with varying drug resistance serving as models for less effective and sensitive responses to doxorubicin, a cell viability test was conducted following 6 and 24 h doxorubicin treatments. The cell lines were uniformly seeded at a concentration of 4000 cells per well in a 96‐well plate. Various concentrations of doxorubicin, ranging from 0.001 to 80 μM, were added to the culture medium, and the cells were incubated for 6 and 24 h. Cell viability was assessed using WST‐8 (Cell Counting Kit‐8; Dojindo Laboratories, Kumamoto, Japan). Absorbance at 450 nm was measured using a cell counter (Sysmex CDA‐500, Sysmex Corporation, Hyogo, Japan).^26^

### 2.3 Confocal microscope

ΔφM and reactive oxygen species (ROS) production were observed using confocal microscopy (Nikon Instruments A1 Confocal Laser Microscope Series With NIS‐Elements C Software). This assessment was conducted considering mitochondrial depolarization as the initial mitochondrial response to drug‐induced stress.^27^ The cells were placed in glass‐bottom dishes with and without 100 nM doxorubicin and treated for 6 h. They were then stained with tetramethyl rhodamine, methyl ester (TMRM) (400 nM, Marker Gene Technologies INC, USA), and Mito Marker Green (MTG) (120 nM, Marker Gene Technologies, Inc, USA) for 30 min. Additionally, The CellROX ROS Detection Kit (1:1000, ab186029, Abcam) was incubated for 45 min in a cell culture incubator. Hoechst 33342 (1:10,000, H3570, Thermo Scientific, USA) was added and incubated for 10 min. The excitation wavelengths for TMRM, MTG, ROS, and Hoechst 33342 were 548, 490, 650, and 361 nm, respectively, while their emission wavelengths were 574, 516, 675, and 486 nm, respectively.

### 2.4 Flow cytometry

ΔφM and mitochondria quantity within the cells were assessed using flow cytometry (BD FACS Aria™ III Cell Sorter). The cells were exposed to TMRM and MTG at concentrations of 400 nM and 120 nM, respectively, and incubated for 30 min. Following harvest through trypsinization and centrifugation (1000 rpm, 4°C, 5 min), the cells were set to a concentration of 1 million cells/mL using a staining buffer. Subsequently, 7‐AAD (10 uL/mL, Bio Legend Way, San Diego) was introduced to ensure the living cells exceeded 85%, and the cell population was promptly assessed using flow cytometry.

### 2.5 Western blot

Western blotting was employed to assess mitochondrial autophagy proteins. Samples were extracted using M‐PER Mammalian Protein Extraction Reagent (Thermo Scientific, USA). Subsequently, the obtained sample liquid was transferred to Amicon Ultra 0.5 mL tubes and centrifuged (14,000×*g*, 30 min). The filtrate was transferred to a new tube, centrifuged (1000×*g*, 2 min) to collect proteins, and then quantified. SDS‐PAGE (12% acrylamide gel) (Bio‐Rad, #1610175) was used with 20 μg of protein per lane, followed by transfer to a 0.2 μm PVDF membrane (Bio‐Rad, USA). The membrane was blocked with 4% skim milk and subsequently incubated overnight at 4°C with primary antibodies: anti‐β‐Actin (Millipore Sigma, #a5441, 1:5000), p62 (Abcam, #ab207305, 1:1000), and anti‐light chain 3B (LC3B) (Abcam, ab18709, 1:1000). After incubation with HRP‐conjugated secondary antibodies, bands were visualized using ECL™ Prime detection reagent (Millipore Sigma), and analysis was conducted using the LAS‐4000 mini‐image analyzer.

### 2.6 Seahorse XF mitochondrial stress assay

The Seahorse XFe96 Analyzer (Agilent, Santa Clara, USA) was used to measure the extracellular acidification rate (ECAR) and oxygen consumption rate (OCR) of cells. Cell plates containing 3 × 10^4^ cells/well were seeded into Cell‐Tak‐coated 96‐well Seahorse plates and pre‐incubated at 37°C without CO2 for 30 min. OCR and ECAR were measured in XF media under basal conditions and in response to specific compounds (2 μM oligomycin, 2 μM carbonyl cyanide‐4 (trifluoromethoxy) phenylhydrazone, 1 μM rotenone + antimycin A) using the Seahorse XFe96 Analyzer.

### 2.7 Patient samples

This retrospective study utilized data from 107 consecutive patients with TNBC who underwent NAC and surgery at two institutions, Tohoku University Hospital and Tohoku Kosai Hospital, both located in Sendai, Japan, between 2015 and 2017. The specimens were obtained before (core needle biopsy) and after receiving NAC. The prognosis information for recurrence‐free survival (RFS) of patients was last updated in January 2023.

### 2.8 Immunohistochemistry (IHC)

Immunohistochemistry (IHC) was performed on 3‐micrometer‐thick sections obtained from paraffin‐embedded samples fixed in 10% formalin. Antigen retrieval for DRP1 was conducted using a pH 6 citrate buffer in a microwave, while p62 antigen retrieval used a pH 9 Tris‐EDTA buffer, and Parkin antigen detection used a pH 8 EDTA buffer. Subsequently, the sections were blocked in 10% rabbit serum (Nichirei Biosciences, Tokyo, Japan). The primary antibodies against DRP1 (Abcam, ab56788, 1:1000), p62 (Abcam, ab207305, 1:2000), and Parkin (Santa Cruz Bio, sc‐32,282, 1:50) were then incubated overnight at 4°C. After blocking endogenous peroxidase activity, the sections were incubated with secondary antibodies (Nichirei Bioscience, Tokyo, Japan). Human kidney, hepatocellular carcinoma (HCC), and gallbladder tissues were used as positive controls for DRP1, p62, and Parkin, respectively.

### 2.9 Assessment of immunoreactivity

Digital analysis software, “HALO TM Area Quantification ver. 2.2 (Indica Labs, Corrales, NM),” was utilized to assess the distinct morphology of tumor cells and their immunoreactivity.^28^ This application initially separated IHC images into hematoxylin and DAB channels. Subsequently, it identified individual cells and graded them based on the intensity of the DAB signal in the cytoplasm. Results generated by HALO included counts and ratios of negative, high, medium, and low‐intensity cells. The total score of IHC staining was determined by multiplying the intensity score (0 = negative, 1 = low, 2 = medium, and 3 = high intensity) by the proportion score, represented as a positive ratio ranging from 0 to 10 (corresponding to 0%–100%), yielding a final score between 0 and 30.^9^ We designated the antibody expression before NAC as “pre” and that after NAC as “post” to differentiate between the characteristics of expression before and after NAC.

### 2.10 Data analysis

All experiments were performed at least thrice. ImageJ was used to digitize the immunofluorescence intensity of confocal microscopy and western blot. The Shapiro–Wilk test analyzed repeated experimental results and the data conformed to a normal distribution. Unpaired t‐test was used to examine the changes in fluorescence intensity of cell lines treated with or without chemotherapy. Ordinary one‐way ANOVA tested the differences of the three cell lines in Seahorse XF mitochondrial stress assay and western blot. Kolmogorov–Smirnov test demonstrated the normal distribution of clinical pathology analysis results. The chi‐square test was used to determine the disparity in protein expression regarding clinical characteristics. Additionally, Cox univariate and multivariate analyses of variables were performed to assess the variables associated with RFS. The closer the Hazard ratio value is to 0, the lower the risk that the former variable of a pair of variables may cause RFS, and the higher the risk that the latter variable may cause RFS. Statistical significance was set at *p* < 0.05. The definition of statistical significance is as follows: *p* <*p < 0.05, **p < 0.01, ***p < 0.001, ****p < 0.0001. Data analyses were conducted employing SPSS V.25 statistical software (Chicago, Illinois, USA) and GraphPad Prism version 9.5.0 for macOS (Boston, Massachusetts USA).

## 3 RESULTS

### 3.1 The doxorubicin‐less effective TNBC cell line‐MDA‐MB‐231 exhibited lower ΔφM

**FIGURE 1:**
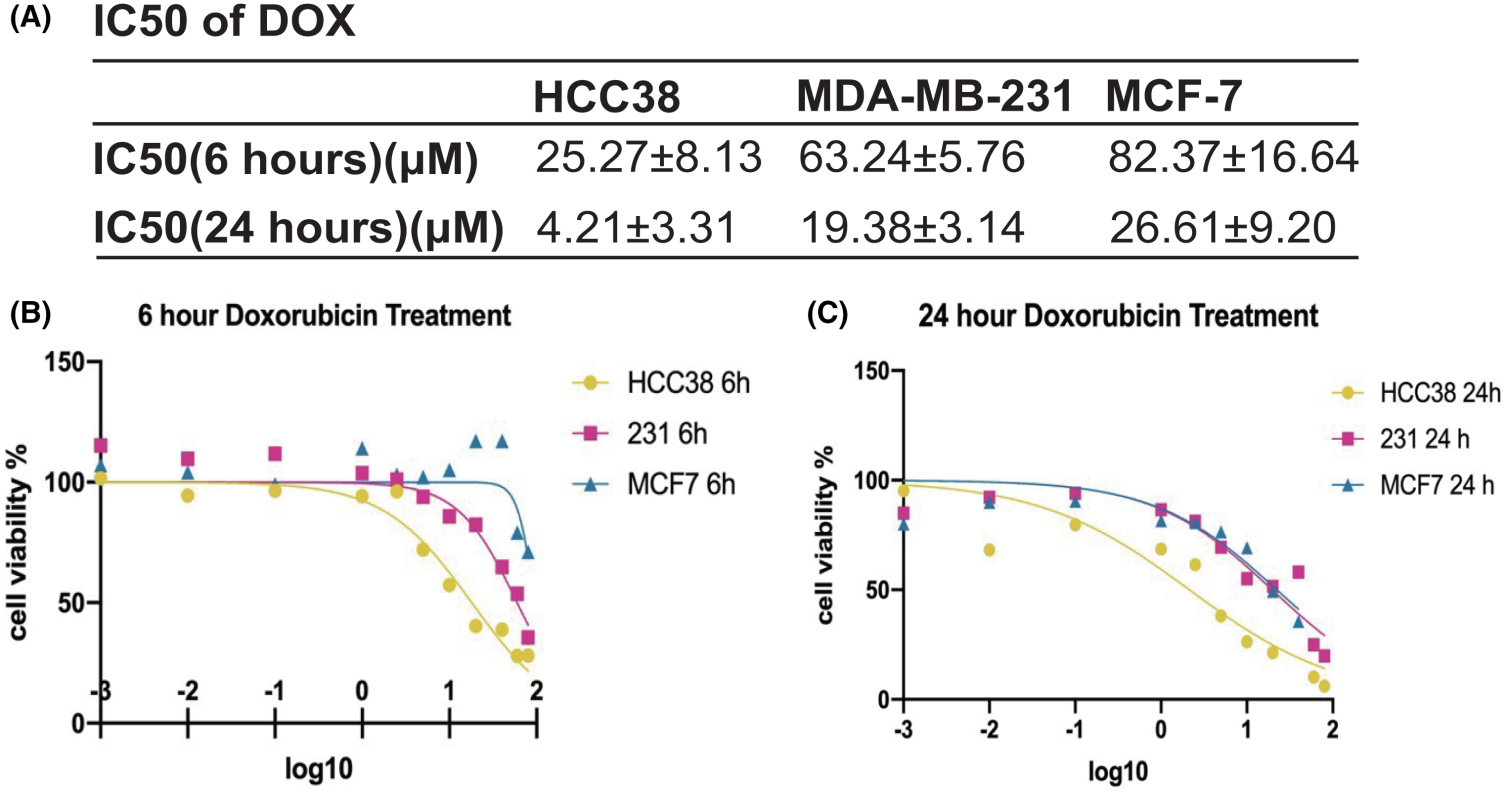
Cell viability assay and IC50. (A) IC50 of doxorubicin in different cell lines. (B) (C) Cell viability assay by using doxorubicin on three cell lines (MDA‐MB‐231, HCC38, and MCF‐7) in 6 and 24 h.

**FIGURE 2:**
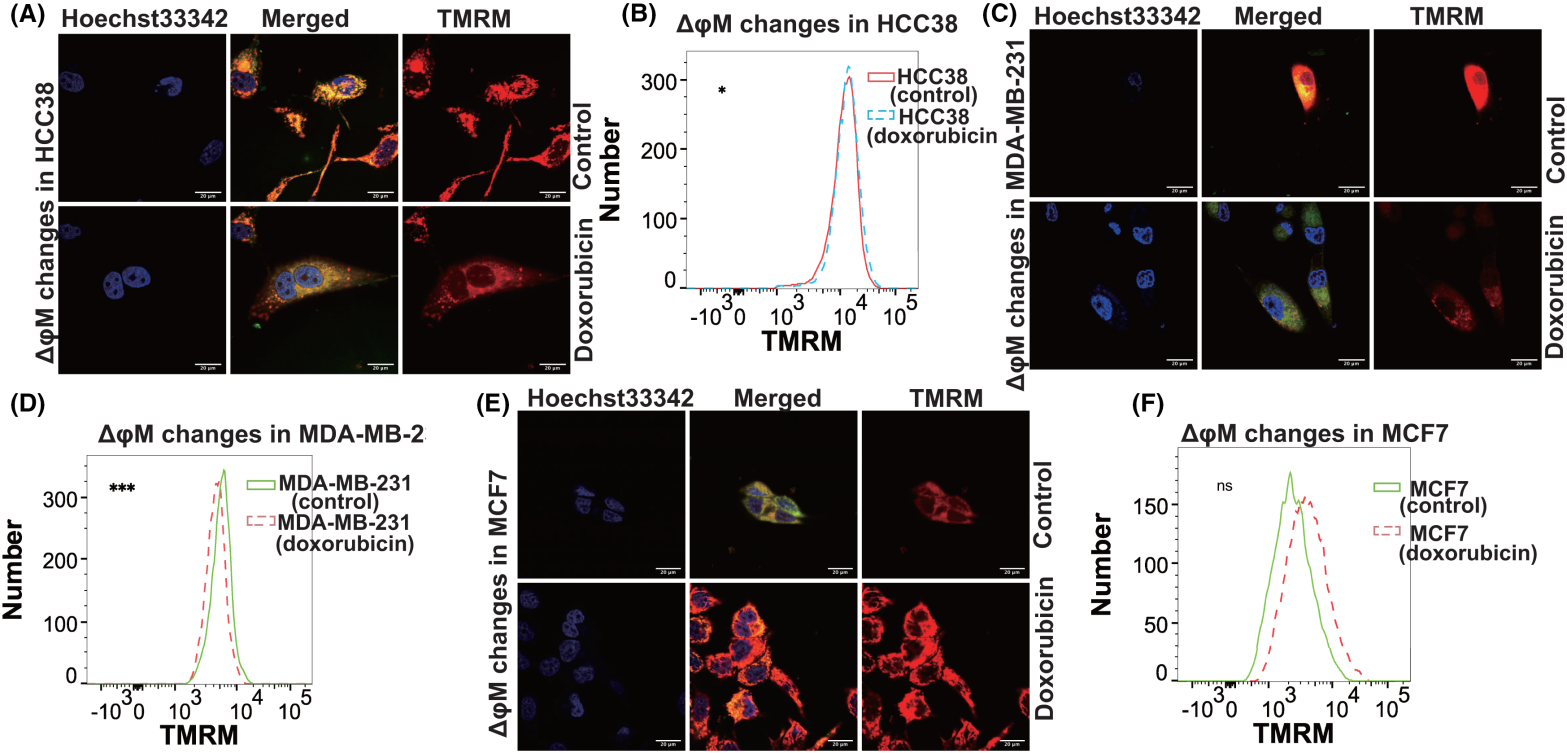
ΔφM of HCC38, MDA‐MB‐231, and MCF7. (A, B) Confocal microscopy images at 100× magnification were captured for HCC38 cells, focusing on ΔφM (control and doxorubicin). Red fluorescence (TMRM) represents ΔφM. MTG was used as a mitochondrial localizer (green). Nuclei were co‐stained using Hoechst 33342 (blue). (B) Flow cytometry analysis of TMRM fluorescence in HCC38 cells (control and doxorubicin‐treated). (C, D) We obtained 100× confocal microscopy images targeting TMRM and performed flow cytometry analysis to measure TMRM fluorescence in MDA‐MB‐231 (control and doxorubicin‐treated). (E, F) We captured 100× confocal microscopy images targeting TMRM and performed flow cytometry analysis to measure TMRM fluorescence in MCF‐7 cells (control and doxorubicin‐treated). An unpaired t‐test was used to ensure the consistency of repeated experimental results.

The cells exhibited varying dose responses to doxorubicin, with HCC38 being most sensitive, followed by MDA‐MB‐231 and MCF‐7, having IC50 values of 4.21 ± 3.3, 19.38 ± 3.14, and 26.61 ± 9.20 μM (Figure 1A). MDA‐MB‐231 and MCF7 exhibited more resistance to doxorubicin treatment than HCC38 (Figure 1B,C). The ΔφM of these three cell lines was assessed using TMRM, a red fluorescence marker, through confocal microscopy and flow cytometry. Results showed a slight increase in ΔφM for HCC38 (Figure 2A,B), while a decrease was observed in MDA‐MB 231 (Figure 2C,D) (*p* = 0.00019, 0.04624, respectively). Simultaneously, the ΔφM of the doxorubicin‐less effective cell line, MCF‐7 (luminal), exhibited an upward trend (*p* = 0.1103) (Figure 2E,F).

### 3.2 MDA‐MB‐231 maintained the stability of ROS with doxorubicin

**FIGURE 3:**
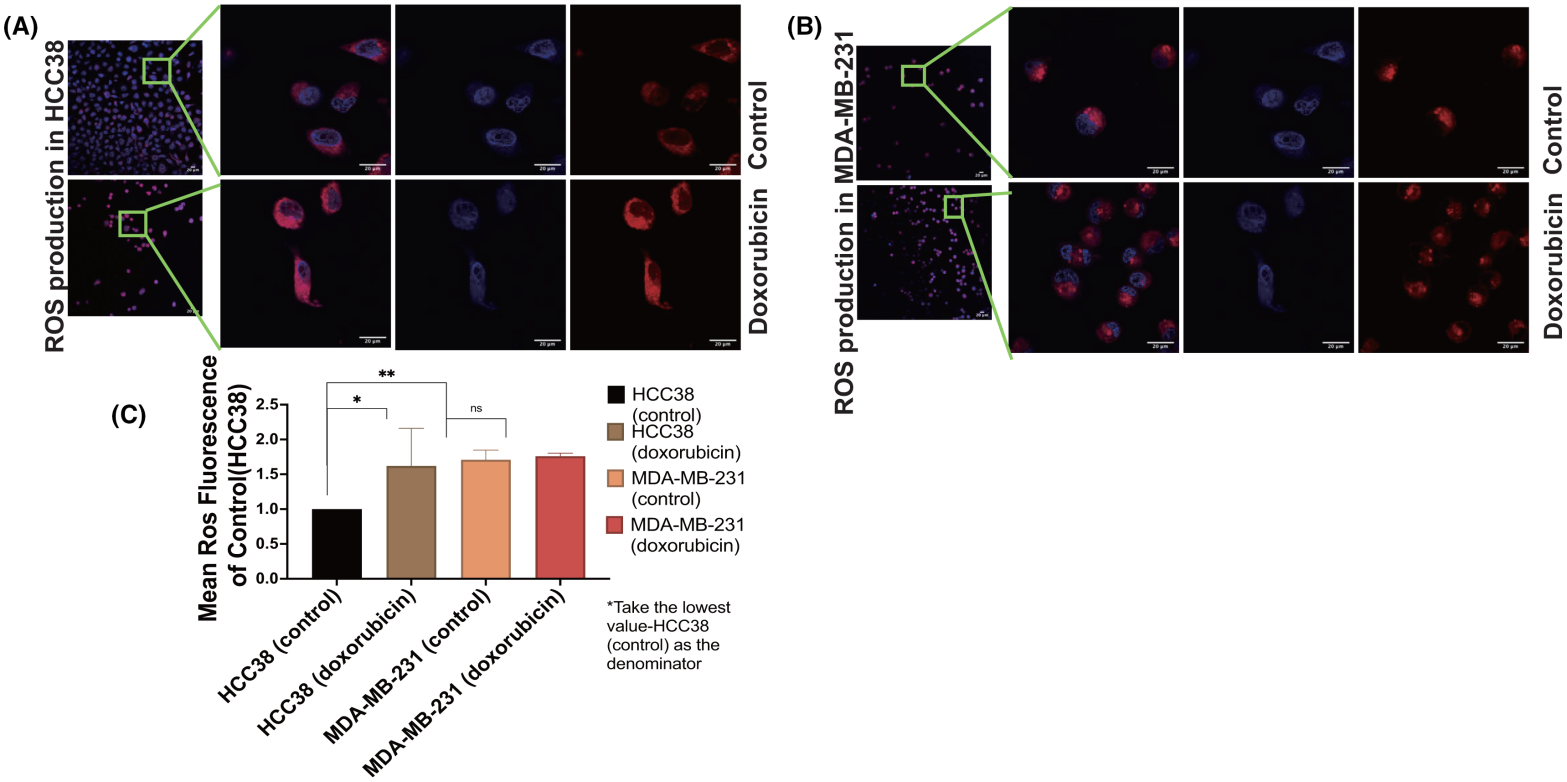
MDA‐MB‐231 maintained the stability of ROS after doxorubicin treatment. (A, B) Images of ROS fluorescence were obtained from HCC38 and MDA‐MB‐231 cells (control and doxorubicin‐treated) under 20× and 100× magnification. Red fluorescence (Ros deep red kit) represents ROS production. Nuclei were co‐stained by Hoechst 33342 (blue). The 20× image captures a large number of cells, reflecting changes in overall ROS production, and the 100× magnification captures representative cells. (C) A histogram was created to display the mean intensity of fluorescence in MDA‐MB‐231 and HCC38 cells, with HCC38 (control) as the denominator using the lowest value as the reference point. An unpaired t‐test was used to ensure the consistency of repeated experimental results.

Subsequently, we assessed ROS production in TNBC cell lines, as superoxide accumulation is one of the mechanisms underlying the anticancer effects of chemotherapy medications.^29^ ROS production significantly increased in HCC38; however, it showed a minimal change in MDA‐MB‐231 after doxorubicin treatment (Figure 3A,B). The results showed that HCC 38 exhibited an increase in ROS levels, whereas MDA‐MB‐231 maintained a consistent ROS level after treatment with doxorubicin. (Figure 3C) (*p* = 0.0350, *p* > 0.05). The baseline ROS level in MDA‐MB‐231 was higher than that in HCC38. This suggests that MDA‐MB‐231 can tolerate cell stress changes for a longer duration and engage in more active signal conduction activities to promote cell proliferation and invasion (Figure 3C) (*p* = 0.0022).^30^

### 3.3 MDA‐MB‐231 utilized mitophagy to maintain ROS stability

**FIGURE 4:**
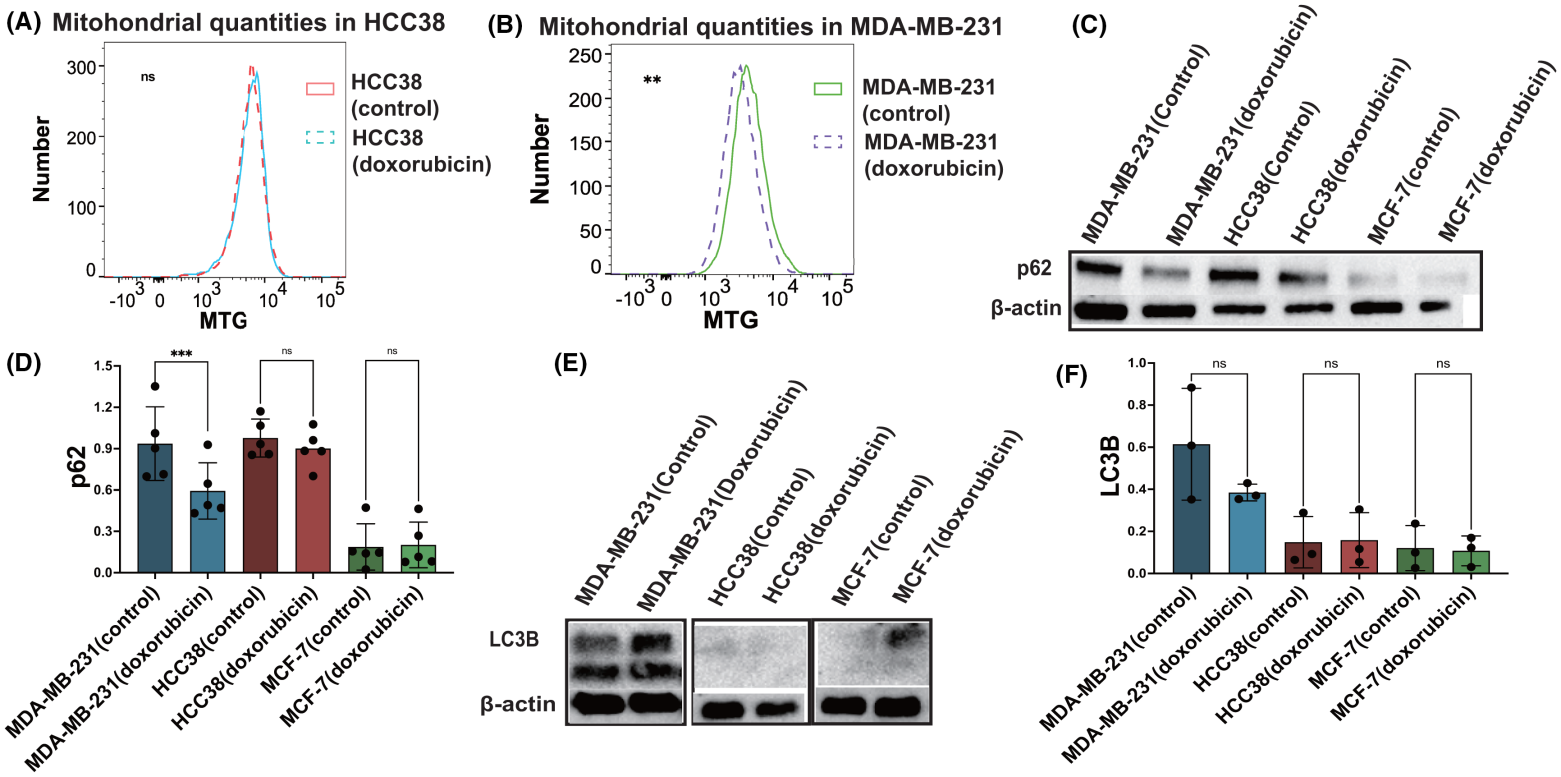
MDA‐MB‐231 specifically used mitophagy to maintain ROS stabilization. (A, B) In flow cytometry, we measured MTG fluorescence in HCC38 and MDA_MB‐231 cells (control and doxorubicin). (C) p62 expression in MDA‐MB‐231, HCC38, and MCF‐7 (control and doxorubicin) by western blotting. (D) Quantification and comparison of p62 expression in three cell lines (control and doxorubicin). (E) LC3B expression in MDA‐MB‐231, HCC38, and MCF‐7 (control and doxorubicin) by western blotting. (F) Quantification of western blots in E. Ordinary one‐way ANOVA was used to ensure the consistency of repeated experimental results.

As the reduced ΔφM is considered to contribute to mitochondrial autophagy, we conducted flow cytometry to assess the mitochondrial quantity. The MTG curve of HCC38 showed minimal changes after treatment with doxorubicin (Figure 4A, *p* > 0.05). In contrast, MDA‐MB‐231 treated with doxorubicin exhibited an increase than the control group, indicating a decrease in mitochondrial content following doxorubicin treatment (Figure 4B, *p* = 0.00691).

The decreased ΔφM and mitochondrial content in MDA‐MB‐231, which were less responsive to the drug, indicates the presence of mitophagy. After mitophagy, P62 binds to LC3B to form autophagosomes and degrades, while LC3B dissociates.^31^ Therefore, we examined mitophagy‐related proteins, specifically LC3B and p62. The results showed that p62 exhibited high expression in TNBC cell lines but was scarcely expressed in MCF7. Furthermore, p62 expression in MDA‐MB‐231 decreased significantly than in HCC38 following exposure to doxorubicin (Figure 4C,D, *p* = 0.0009). LC3B exhibited significantly high expression levels exclusively in MDA‐MB‐231 (Figure 4E). After doxorubicin treatment, LC3B expression increased slightly (n.s) (Figure 4F). These findings suggest that MDA‐MB‐231 specifically utilizes mitochondrial autophagy to alleviate excessive ROS pressure, potentially leading to drug resistance.

### 3.4 Less effective cell line doxorubicin relies on OXPHOS metabolism, whereas doxorubicin‐sensitive cell lines depend on glycolysis

**FIGURE 5:**
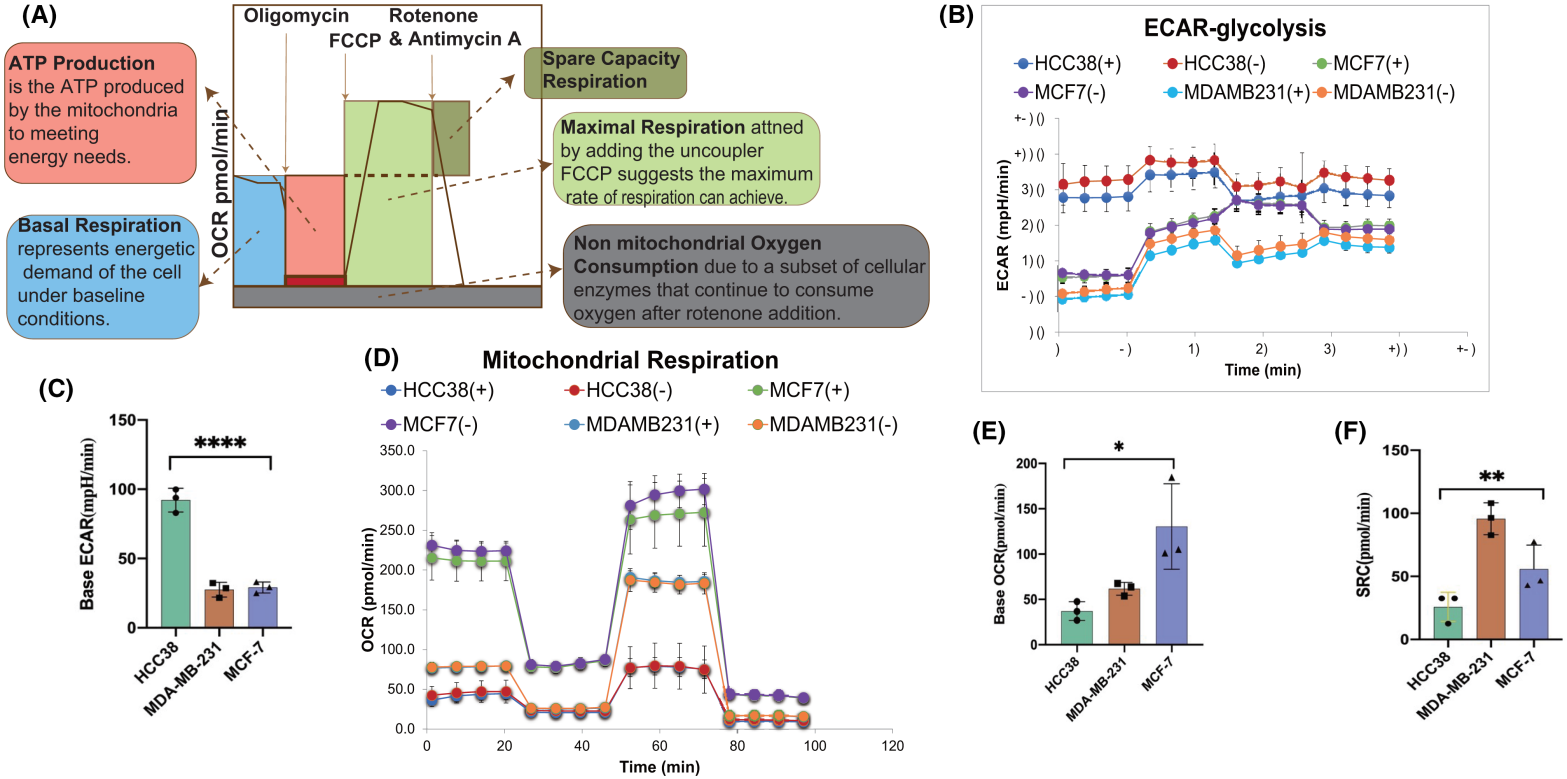
The cell line less responsive to doxorubicin primarily relies on OXPHOS metabolism, in contrast to HCC38. (A) Interpretation of the components of the Seahorse XF cell mitochondrial stress assay. (B, C) ECAR‐glycolysis and OCR‐mitochondrial respiration (OXPHOS) in three cell lines, both with (+) and without (−) doxorubicin injection. (D) Histogram of basal ECAR in three cell lines. Basal ECAR of HCC38 is the highest (*p* < 0.0001). (E) Histogram of basal OCR in three cell lines. The basal OCR of HCC38 is the lowest (*p* = 0.0162). (F) Histogram of SRC in three cell lines. SRC of HCC38 is the lowest (*p* = 0.0034). An ordinary one‐way ANOVA was used to ensure the consistency of repeated experimental results.

MDA‐MB‐231, a cell line with poor responsiveness to doxorubicin, was found to maintain stable levels of ROS through mitochondrial autophagy. Considering the correlation between these mechanisms and cellular metabolic phenotypes, we hypothesized that these cell lines would exhibit distinct metabolic characteristics.^32^, ^33^ The Seahorse XFe96 Analyzer was used to measure oxidative phosphorylation (OXPHOS) through OCR and glycolysis through ECAR (Figure 5A).^34^ Spare respiratory capacity (SRC) represents an indicator of cellular responsiveness to energy demands or cellular health. Figure 5A displays additional metrics for better understanding.

The results showed that the initial ECAR of HCC38 was significantly higher than that of the other two cell lines (Figure 5B,C, *p* < 0.001). Conversely, the base OCR of MDA‐MB‐231 and MCF‐7 exceeded that of HCC38 (Figure 5D,E, *p* = 0.0162). The SRC of HCC38 was significantly lower than that of others (*p* < 0.005) (Figure 5F). Doxorubicin treatment had no significant effect on metabolic changes in all cell lines.

Therefore, considering the changes in ΔφM, mitophagy conditions, and metabolism features, HCC38 exhibited sensitivity to doxorubicin primarily through glycolysis, while doxorubicin‐less effective cells, MDA‐MB‐231, and MCF‐7 mainly use OXPHOS for metabolism. These findings reveal mitophagy combined with OSPHOS metabophenotype to mitigate excess ROS stress. This phenomenon may have contributed to NAC resistance specifically observed in TNBC.

### 3.5 Mitophagy‐related proteins were expressed in TNBC

The study has demonstrated a unique coexistence of mitophagy and OXPHOS as a mechanism that enables drug resistance to external pressure in TNBC cell lines, which were less responsive to treatment. Therefore, clinicopathological studies were conducted to examine the relationship between mitochondrial dynamic features and in vitro clinical aspects. DRP1 induces mitochondrial fission while Parkin, a component of the PINK1/Parkin‐mediated pathway, functions as a tumor suppressor. P62 is packaged with mitochondria into mitolysosomes.^35^ Therefore, these three proteins were assessed in 107 patients.

**FIGURE 6:**
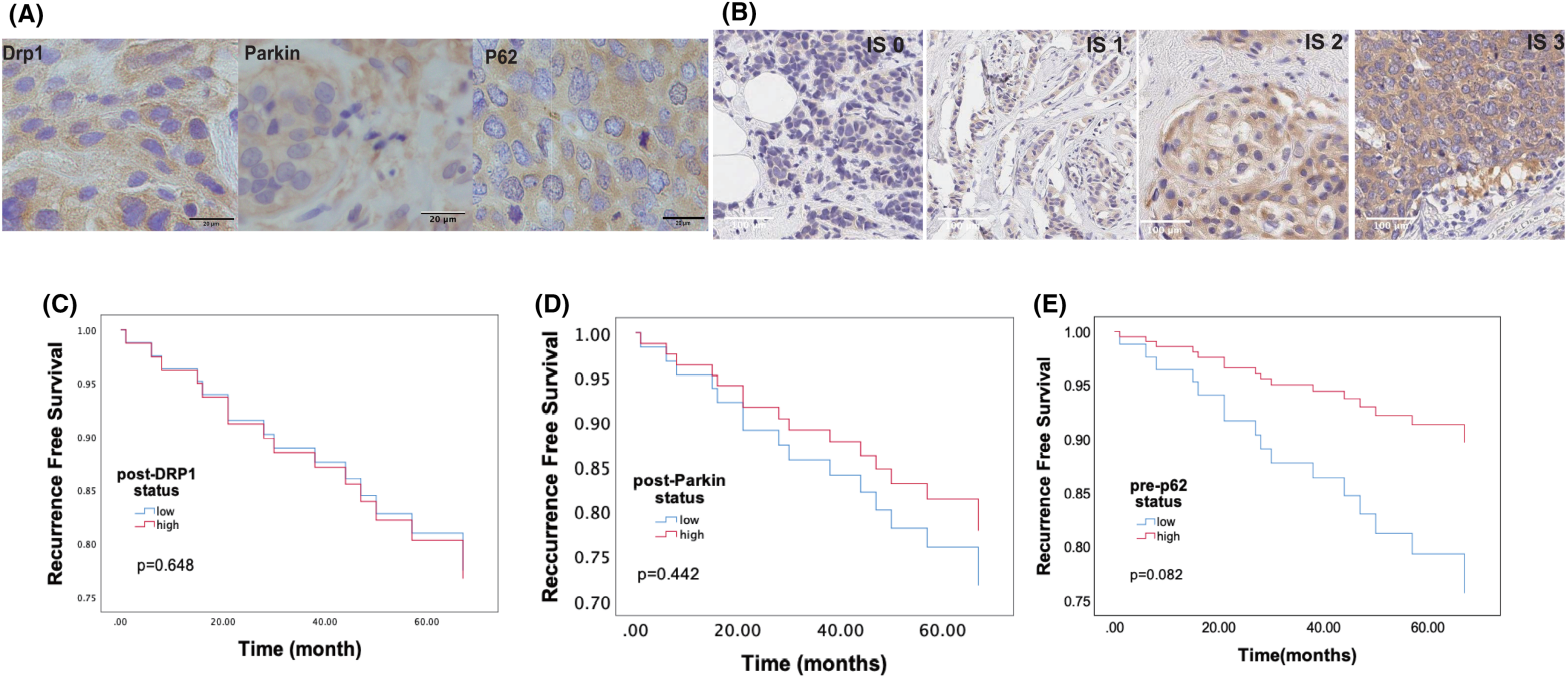
Mitophagy‐related proteins are highly expressed in TNBC patients. (A) IHC images of positive controls of DRP1 (kidney), Parkin (gall bladder), and p62 (HCC). (B) IHC images of four intensity scores. (C–E) RFS with pre‐DRP1, pre‐p62, and pre‐Parkin expression. The data were analyzed using the chi‐square test and Cox regression analysis.

Seventy‐seven patients (78%) had lymph node metastasis, and 96 patients (86.7%) had received anthracyclines (Table 1). Thirty‐one patients (29%) achieved pathological complete response (Grade 3, pCR) after NAC (Table 2). FDG‐PET is a potent imaging tool employed for assessing tumor glycolysis and FDG uptake, which is quantified as the maximum standardized uptake value (SUVmax). This technique is commonly utilized to assess cancer aggressiveness.^36^ Among patients who underwent FDG‐PET after NAC and had a post‐SUV max value <2.5, there was a notably high non‐pCR rate of 60.5%. This suggests that FDG‐PET assessment after NAC may not be a reliable predictor of pCR (Table 2). Figure 6A shows the representative images of DRP1, p62, and Parkin expression. Figure 6B shows the IHC images of four intensity scores. We categorized the total scores into high and low‐expression groups, using a cutoff of 10.5. The results indicated that pre‐DRP1 exhibited high expression levels in specific patient groups, including those under 50 years of age (<50), individuals with ER‐/PR‐ (HR‐) status, patients with TNBC, and those with a high Ki‐67 (20% cutoff) rate (*p* = 0.002, 0.031, 0.013, 0.015, respectively) (Table 3). No correlation was observed between pre‐/post‐DRP1 expression and the NAC effect (Tables 3 and 4). Pre‐Parkin was also expressed in HR‐ (Table 3, *p* = 0.034). We performed a multivariable analysis on factors that demonstrated *p*‐values below 0.2 in the univariable analysis. Pre‐DRP1 and pre‐Parkin were not risk factors for RFS in patients with breast cancer (Figure 6C,D). Low expression of pre‐P62 showed a higher risk of RFS than high pre‐p62 expression, although this was not significant (Figure 6E; Table 5) (HR = 2.459, *p* = 0.082).

**TABLE 1:** Clinicopathological characters of the patients.

Factors	Number	Percentage (%)
Median age (years)	54.55 (30–79)	
cT status		
1/2/3/4	20/69/5/14	17.8/ 64.5/ 4.7/ 13.1
cN status		
N0/1/2/3	30/59/8/10	28.0/ 55.1/ 7.5/ 9.4
cStage status		
I/II/III	8/71/28	7.5/ 66.4/ 26.7
Pre subtype		
ER+ HER2‐	41	38.3
ER+ HER2+	17	15.9
HER2 enriched	18	16.8
TNBC	31	29
Median Pre Ki‐67	49% (6–91%)	
Regimen of chemotherapy		
Anthracycline and Taxane	69	64.5
Anthracycline, Taxane and Trastuzmab ± Pertuzumab	27	25.2
Others or unknown	11	10.3
FDG‐PET		
Before NAC	76	71
After NAC	65	60.7

Abbreviations: cN, clinical Node; cT, clinal Tumor; ER, estrogen receptor; FDG‐PET, 2‐deoxy‐2‐[fluorine‐18] fluoro‐D‐glucose integrated with computed tomography; HER2, human epidermal growth factor receptor 2; NAC, neoadjuvant chemotherapy; TNBC, triple negative.

**TABLE 2:** Comparison of assessments between pathologic response and FDG‐PET.

	Non‐pCR		pCR		Total	*p* Value
	*n*	%	*n*	%		
Total	76	71.0	31	29.0	107	
Pre subtype						
ER + HER2+	15	88.2	2	11.8	17	<0.0001****
ER + HER2‐	35	85.4	6	14.6	41	
HER2 enriched	18	42.1	11	57.9	19	
TNBC	19	61.3	12	38.7	31	
SUV max						
Pre‐SUVmax ≥ 10	24	70.6	10	29.4	34	0.884
Pre‐SUVmax < 10	29	69.0	13	31.0	41	
Post‐SUVmax ≥ 2.5	18	81.8	4	18.2	22	0.036*
Post‐SUVmax < 2.5	26	60.5	17	39.5	43	
Tumor size						
>5 cm	15	78.9	4	21.1	19	0.401
≤5 cm	61	69.3	27	30.7	88	
Nodal status						
N0	19	63.3	11	36.8	30	0.273
N+	57	74	20	26	77	
Ki‐67 (20%)						
High	57	66.3	29	33.7	86	0.034*
Low	14	93.3	1	6.7	15	

Abbreviations: ER, estrogen receptor; HER2, human epidermal growth factor receptor 2; pCR, pathological complete response; post, after chemotherapy; pre, before chemotherapy; SUVmax, maximum standardized uptake value; TNBC, triple negative.

*p < 0.05, **p < 0.01, ***p < 0.001, ****p < 0.0001

**TABLE 3:** Clinicopathological features and association with the status of pre‐DRP1, p62, and Parkin.

	Pre‐DRP1		*p* Value	Pre‐p62		*p* Value	Pre‐Parkin		*p* Value
	High	Low		High	Low		High	Low	
Age (years)									
<50	25	39	0.002**	25	15	0.872	19	20	0.556
≥50	28	12		41	23		35	29	
Tumor size									
>5 cm	4	14	0.007**	11	7	0.82	10	8	0.945
≤5 cm	48	36		55	31		44	41	
Nodal status									
+	37	38	0.593	47	28	0.787	38	36	0.727
−	16	13		19	10		16	13	
HR+	33	36	0.031*	31	26	0.034*	24	32	0.034*
HR−	32	15		35	12		30	17	
TNBC	21	9	0.013*	20	10	0.666	16	14	0.906
Non TNBC	32	42		46	28		38	35	
Ki67 (20%)									
High	48	37	0.015*	59	26	0.015*	47	38	0.064
Low	3	11		5	9		4	10	
Pathological response grade									
1	21	31	0.097	32	20	0.549	26	25	0.729
2	13	8		12	9		10	11	
3	19	12		22	9		18	13	

Abbreviations: DRP1, Dynamin‐related protein 1; HR, hormone receptor; p62, Sequestosome 1; pre, before chemotherapy; TNBC, triple negative breast cancer.

*p < 0.05, **p < 0.01, ***p < 0.001, ****p < 0.0001

**TABLE 4:** Clinicopathological features and association with the status of post‐DRP1, p62, and Parkin.

	Post‐DRP1		*p* Value	Post‐p62		*p* Value	Post‐Parkin		*p* Value
	High	Low		High	Low		High	Low	
Age (years)									
<50	13	13	0.25	15	11	0.644	14	11	0.881
≥50	17	30		25	23		26	22	
Tumor size									
>5 cm	9	6	0.095	9	6	0.605	7	8	0.478
≤5 cm	21	37		31	28		33	25	
Nodal Status									
+	23	31	0.488	31	24	0.379	29	26	0.565
−	6	12		8	10		10	7	
HR+	19	28	0.876	27	21	0.607	24	24	0.254
HR−	11	15		13	13		16	9	
TNBC	7	12	0.661	9	10	0.498	10	8	0.94
Not TNBC	23	31		31	24		30	25	
Ki67 (20%)									
High	26	29	0.036	30	26	0.986	31	25	0.549
Low	2	11		7	6		6	7	

Abbreviations: DRP1, Dynamin‐related protein 1; HR, hormone receptor; p62, Sequestosome 1; post, after chemotherapy; TNBC, triple negative breast cancer.

**TABLE 5:** Univariate and multivariate analyses of variables associated with RFS in TNBC.

Variables	Univariable			Multivariable		
	Hazard ratio	95% CI	*p* Value	Hazard ratio	95% CI	*p* Value
Age (<50 vs. ≥50)	0.89	0.32–2.45	0.817			
Tumor size (≤5 cm vs. >5 cm)	0.64	0.21–2.00	0.447			
Nodal status (− vs. +)	1.29	0.45–3.71	0.642			
HR− vs. HR +	0.50	0.18–1.43	0.197	1.18	0.37–3.78	0.778
TNBC (no vs. yes)	0.83	0.33–2.66	0.898			
Ki67 (<20% vs ≥ 20%)	0.90	0.20–4.02	0.895			
pCR vs. not pCR	0.15	0.02–1.17	0.07	0.01	0.02–1.12	0.063
Pre‐DRP1 (low vs. high)	1.26	0.47–3.39	0.648			
Pre‐p62 (low vs. high)	2.46	0.92–6.61	0.074	2.55	0.89–7.34	0.082
Pre‐Parkin (low vs. high)	0.65	0.23–1.84	0.422			
Post‐DRP1 (low vs high)	0.96	0.34–2.72	0.94			
Post‐p62 (low vs. high)	0.87	0.31–2.46	0.798			
Post‐Parkin (low vs. high)	1.33	0.48–3.67	0.584			

Abbreviations: CI, confidence interval; DRP1, Dynamin‐related protein 1; HR, hormone receptor; p62, Sequestosome 1; pCR, pathological complete response; post, after chemotherapy; pre; before chemotherapy; TNBC, triple negative breast cancer.

## 4 DISCUSSION

Despite significant progress in breast cancer screening, diagnosis, and treatment, TNBC remains a formidable challenge for clinical oncologists. This is primarily attributed to the unfavorable patient outcomes associated with TNBC. Conventional therapeutic approaches, including endocrine and anti‐HER2 therapies, which are often effective in treating other types of BC, proved ineffective in TNBC owing to the absence of hormone receptors and HER2 amplification. Therefore, chemotherapy is the primary therapeutic approach for TNBC treatment. NAC is a proven treatment for high‐risk, locally advanced, or unresectable breast cancers that allows the evaluation of treatment response and enables the discontinuation of treatment in cases with tumor progression. Moreover, pCR is used as a surrogate marker for disease‐free survival and overall survival.^37^ Among patients with breast cancer treated with NAC, TNBC has the most favorable pCR rate compared to other subtypes.^38^ Furthermore, previous studies have shown that the pCR rate in TNBC is most significantly correlated with its prognosis. Consequently, it is essential to investigate sensitivity to NAC treatment.^39^ Programmed death ligand 1 (PD‐L1) is gaining wide interest in TNBC, with a positivity rate of 20%–60%. However, approximately 30% of patients with TNBC still achieve non‐pCR, with a significant probability of recurrence.^3^, ^40^ Focusing on mitochondrial metabolic signatures associated with tumors could offer additional therapeutic options for this aggressive BC subtype. This is particularly relevant because metabolism is recognized as one of the hallmarks of cancer. However, metabolic‐based therapy, other than the progress in the treatment of glycolytic subtypes, provides less information.^41^

In our study, we found through cell experiments that the less effective TNBC cell line MDA‐MB‐231‐specific mitophagy combined with OXPHOS metabolism resisted the therapeutic effect of doxorubicin. This result has also been shown in clinical pathological analysis and has certain significance which firstly demonstrated the unique contribution of high mitochondrial metabolism with mitophagy to TNBC drug resistance from the perspective of FDG‐PET limitations and pilot studies of mitochondrial metabolites. We also suggested the significance of detecting and evaluating mitochondrial metabolism, mitochondrial fission in TNBC patients and mitophagy related to worse prognosis, provided theoretical support for the next study of OSPHOS‐tracer‐PET/mitochondrial autophagy tracer‐PET.

Doxorubicin induces iron chelation, leading to a rapid increase in ROS levels and thus accelerating the death of cancer cells.^42^ The rapid increase of ROS in HCC38 and the stable maintenance of ROS in MDA‐MB‐231 suggest that HCC38 is sensitive and MDA‐MB‐231 is less effective to doxorubicin. Metabolic analysis revealed that doxorubicin‐less effective cell lines are dependent on OXPHOS. Another study also found that after glycolysis is inhibited, glucose metabolism is transferred to OXPHOS in an autophagy‐dependent manner, which promotes cell survival, thus confirming our results.^43^ Chemotherapy had little effect on metabolic status; however, combined with intracellular ROS production, we observed ΔφM, mitochondrial loss, and specific expression of LC3B and p62, which indicated that the coexistence of mitophagy and OXPHOS avoids drug intervention only in the doxorubicin‐resistant TNBC cell line. The resistance mechanism of the luminal cell line is independent of mitophagy.

Subsequently, we performed an IHC analysis to investigate the potential association between mitochondrial fission, canonical mitophagy, and drug sensitivity. The limitations of FDG‐PET in predicting pCR highlight its inability to assess non‐glycolytic metabolism in BC. DRP1 is highly expressed in TNBC, indicating mitochondrial fission and low p62 expression seems to be a high‐risk factor for RFS, indicating mitophagy with a poor prognosis. DRP1 regulates mitochondrial fission, which plays a crucial role in the initiation of mitophagy. This process encompasses both canonical and receptor‐mediated pathways.^35^ Receptor‐mediated mitophagy, which involves proteins such as FUNDC1, BBNIP3, and BNIP3L/NIX, is associated with treatment in cancer cells and prognosis. Mitophagy controlled by BNIP3L pathways protects glioblastoma cells from lack of oxygen.^44^, ^45^ Therefore, the overexpression of DRP1 induces mitochondrial fission, leading to a decrease in ΔφM levels when mitochondria are under stress, thereby initiating mitochondrial autophagy. In the context of fragmented and damaged mitochondria, phosphatase and tensin homolog‐induced kinase 1 (PINK1) recruits Parkin. Subsequently, the adaptor protein p62 recognizes phosphorylated polyubiquitin chains and initiates the formation of the mitochondrial autophagosome by binding to microtubule‐associated protein LC3.^35^ The classical pathway of PINK1‐Parkin‐mediated mitochondrial autophagy is named after juvenile Parkinson's disease. It is noteworthy that Parkin often functions as a tumor suppressor.^46^, ^47^ The upregulation of PINK1 in lung and esophageal cancers indicates classical mitochondrial autophagy associated with chemotherapy resistance.^47^, ^48^ Both mitochondrial autophagy pathways are implicated in the therapeutic resistance of hepatocellular carcinoma.^20^, ^43^ These findings align with the phenomenon of mitochondrial autophagy observed in TNBC cells. Our study underscores oxidative phosphorylation as a primary metabolic signature and elucidates the role of mitochondrial autophagy in cancer development.

Our study had some limitations. First, our understanding of the impact of mitophagy on breast cancer, including both the canonical and other mitophagy pathways, remains insufficient. In the future, it will be essential to conduct more experiments using mitophagy‐related gene knockout and amplification cell models. These experiments will assist in identifying targets for monitoring mitophagy, mitochondrial metabolism, and potential therapies. In addition, We have not yet identified suitable targets for PET or other imaging and non‐invasive detection methods in patients with mitochondrial metabolism. This remains one of our future research objectives. If feasible, we will also identify the medications required to alter the metabolic state when mitophagy or a prolonged OXPHOS metabolic state is present. Finally, in pathological clinical analysis, it is challenging to eliminate the influence of different kinds of drugs used in NAC regimens on the expression of these proteins. Exploring whether mitophagy is involved in the response of tumors to other drugs remains a subject that requires ongoing investigation.

## 5 CONCLUSIONS

TNBC cells possess the capacity for efficient mitochondrial autophagy while relying on oxidative phosphorylation for metabolism, enabling resistance to and survival during chemotherapy. In contrast, cells dependent on glycolysis are unable to initiate mitochondrial autophagy to counteract the high levels of ROS generated by chemotherapy, ultimately leading to cell death. Collectively, we have demonstrated a high occurrence of mitochondrial autophagy in TNBC cell lines, which is associated with an unfavorable prognosis in breast cancer. In the future, we hope to conduct in‐depth studies to investigate multiple mitophagy pathways. This is necessary to explore novel therapeutic options that target the unique mitochondria metabolic signatures within tumor cells.

## AUTHOR CONTRIBUTIONS

**Yuechen Wang:** Data curation (lead); formal analysis (lead); investigation (lead); methodology (equal); validation (equal); writing – original draft (equal); writing – review and editing (equal). **Narumi Harada‐shoji:** Funding acquisition (lead); project administration (lead); supervision (lead); validation (lead); writing – review and editing (lead). **Narufumi Kitamura:** Methodology (equal); supervision (equal). **Yuto Yamazaki:** Methodology (equal); resources (equal); supervision (equal). **Akiko Ebata:** Resources (equal). **Masakazu Amari:** Methodology (equal); resources (equal). **Mika Watanabe:** Project administration (equal); resources (equal). **Minoru Miyashita:** Methodology (equal); writing – review and editing (equal). **Hiroshi Tada:** Supervision (equal); visualization (equal). **Takaaki Abe:** Methodology (equal); resources (equal); supervision (equal). **Takashi Suzuki:** Methodology (equal); resources (equal); supervision (equal). **Kohsuke Gonda:** Methodology (equal); project administration (equal); supervision (equal). **Takanori Ishida:** Project administration (equal); supervision (equal); writing – review and editing (equal).

## CONFLICT OF INTEREST STATEMENT

The authors have no conflict of interest.

## ETHICS STATEMENT

This study was approved by institutional review boards and the ethical committees in Tohoku University Hospital (No. 2020‐1‐448) and Tohoku Kosai Hospital (kkrtohoku‐202205brea_S1_01). The researchers received informed permission from participants using an opt‐out procedure on the website. Individuals that declined participation were subsequently excluded.

## CLINICAL TRIAL REGISTRATION NUMBER

We have not conducted clinical trials.

## Data Availability

The data that support the findings of this study are available on request from the corresponding author. The data are not publicly available due to privacy or ethical restrictions.
